# Repurposing and Reformulation of the Antiparasitic Agent Flubendazole for Treatment of Cryptococcal Meningoencephalitis, a Neglected Fungal Disease

**DOI:** 10.1128/AAC.01909-17

**Published:** 2018-03-27

**Authors:** Gemma L. Nixon, Laura McEntee, Adam Johnson, Nicola Farrington, Sarah Whalley, Joanne Livermore, Cristien Natal, Gina Washbourn, Jaclyn Bibby, Neil Berry, Jodi Lestner, Megan Truong, Andrew Owen, David Lalloo, Ian Charles, William Hope

**Affiliations:** a Department of Chemistry, University of Liverpool, Liverpool, United Kingdom; b Antimicrobial Pharmacodynamics and Therapeutics, Department of Molecular and Clinical Pharmacology, Institute of Translational Medicine, Liverpool, United Kingdom; c ithree Institute, University of Technology Sydney, Sydney, Australia; d Department of Molecular and Clinical Pharmacology, Liverpool, United Kingdom; e Liverpool School of Tropical Medicine, Liverpool, United Kingdom; f Quadram Institute Bioscience, Norwich Research Park, Norwich, United Kingdom

**Keywords:** Cryptococcus neoformans, cryptococcal meningoencephalitis, benzimidazole, flubendazole, β-tubulin, antifungal agents, cryptococcal, meningitis, pharmacodynamics, pharmacokinetics, tubulin

## Abstract

Current therapeutic options for cryptococcal meningitis are limited by toxicity, global supply, and emergence of resistance. There is an urgent need to develop additional antifungal agents that are fungicidal within the central nervous system and preferably orally bioavailable. The benzimidazoles have broad-spectrum antiparasitic activity but also have *in vitro* antifungal activity that includes Cryptococcus neoformans. Flubendazole (a benzimidazole) has been reformulated by Janssen Pharmaceutica as an amorphous solid drug nanodispersion to develop an orally bioavailable medicine for the treatment of neglected tropical diseases such as onchocerciasis. We investigated the *in vitro* activity, the structure-activity-relationships, and both *in vitro* and *in vivo* pharmacodynamics of flubendazole for cryptococcal meningitis. Flubendazole has potent *in vitro* activity against Cryptococcus neoformans, with a modal MIC of 0.125 mg/liter using European Committee on Antimicrobial Susceptibility Testing (EUCAST) methodology. Computer models provided an insight into the residues responsible for the binding of flubendazole to cryptococcal β-tubulin. Rapid fungicidal activity was evident in a hollow-fiber infection model of cryptococcal meningitis. The solid drug nanodispersion was orally bioavailable in mice with higher drug exposure in the cerebrum. The maximal dose of flubendazole (12 mg/kg of body weight/day) orally resulted in an ∼2 log_10_CFU/g reduction in fungal burden compared with that in vehicle-treated controls. Flubendazole was orally bioavailable in rabbits, but there were no quantifiable drug concentrations in the cerebrospinal fluid (CSF) or cerebrum and no antifungal activity was demonstrated in either CSF or cerebrum. These studies provide evidence for the further study and development of the benzimidazole scaffold for the treatment of cryptococcal meningitis.

## INTRODUCTION

Cryptococcal meningoencephalitis (here referred to as meningitis) is a common and lethal disease in immunosuppressed patients (1, 2). This disease is predominately associated with advanced HIV infection and has the highest incidence in low- to middle-income countries (1). The number of effective agents is despairingly small (3). All available induction and maintenance regimens are constructed with three antifungal agents: amphotericin B (AmB), flucytosine (5FC), and fluconazole (4). Each of these compounds has significant adverse effects that include infusional toxicity (AmB), nephrotoxicity (AmB [5]), bone marrow suppression (AmB and 5FC [5, 6]), and hepatotoxicity (fluconazole and 5FC [7]). Moreover, there are significant inherent limitations that include fungistatic effects (fluconazole [8]) and the potential emergence of drug resistance (fluconazole and 5FC [9–11]). Thus, there is an urgent imperative to develop new agents. Orally bioavailable agents are particularly important given the predominance of this disease in resource-constrained settings.

During the process of screening a compound library against fungal pathogens, it was noted by us (Megan Truong and Ian Charles) that flubendazole has potent *in vitro* activity against Cryptococcus neoformans. A literature search revealed other members of the benzimidazole class (e.g., albendazole and mebendazole) of antiparasitic agents had previously been demonstrated to have potent *in vitro* activity against Cryptococcus neoformans, with MICs of 0.16 to 0.45 mg/liter (12, 13). The pharmacological target of the benzimidazoles against Cryptococcus neoformans is β-tubulin (14). The antifungal activity of parenterally administered flubendazole in a murine model of cryptococcal meningitis was confirmed by us in a series of preliminary experiments. Concurrently, we became aware of the efforts by Janssen Pharmaceutica to develop a new orally bioavailable formulation of flubendazole that may be active against filariasis and onchocerciasis. The potential value of this new formulation as an oral medicine for the treatment of cryptococcal meningitis in resource-poor health care settings was therefore evident.

Here we describe the *in vitro* activity, putative structure-activity relationships, and *in vivo* pharmacokinetic-pharmacodynamic (PK-PD) relationships of flubendazole against Cryptococcus neoformans. A hollow-fiber infection model of cryptococcal meningitis was developed as a first step for exploring dose exposure-response relationships. Subsequently, two extensively used and well-characterized laboratory animal models of cryptococcal meningitis were used to provide the experimental foundation for the potential use of oral formulations of flubendazole or its congeners for the treatment of a neglected infection of global significance.

## RESULTS

### *In vitro* studies.

Flubendazole displayed potent *in vitro* activity (MICs of 0.06 to 0.25 mg/liter [Table 1]) against C. neoformans. The MICs were comparable when European Committee on Antimicrobial Susceptibility Testing (EUCAST) and Clinical and Laboratory Standards Institute (CLSI) methodologies were used.

**TABLE 1:** MIC distributions of flubendazole against C. neoformans isolates using CLSI and EUCAST methodologies

Methodology	No. of strains	No. of isolates with MIC (mg/liter) of:				
		0.03	0.06	0.125	0.25	0.5
EUCAST	50	1	19	25	5	0
CLSI	50	2	40	8	0	0

The flubendazole 50% inhibitory concentration (IC_50_) against porcine tubulin was 2.38 μM. Other known tubulin inhibitors display similar efficacies in this assay (e.g., colchicine has an IC_50_ of 1.15 μM [unpublished data], paclitaxel has an IC_50_ of 3.9 μM [15], and vinblastine has an IC_50_ of 5.3 μM [15]). These data are consistent with the known mechanism of action of flubendazole.

An *in vitro* drug metabolism and pharmacokinetics (DMPK) assessment of commercially available flubendazole powder confirmed a favorable log of distribution coefficient at pH 7.4 (logD7.4) of 2.9. Plasma protein binding was 90.6%, and there was low metabolic turnover (human microsomal intrinsic clearance = 44 μl/min/mg and rat hepatic intrinsic clearance = 39 μl/min/10^6^ cells). However, aqueous solubility was poor (0.8 μM), which is characteristic of the benzimidazoles. This *in vitro* DMPK assessment was consistent with subsequent *in vivo* observations (see below). Poor aqueous solubility limits absorption through the gut, but once in the bloodstream, flubendazole has favorable pharmacokinetic properties (e.g., ability to pass through cell membranes, low metabolism, and high concentrations of free drug) that enable it to reach the effect site.

### Docking studies.

**FIG 1:**
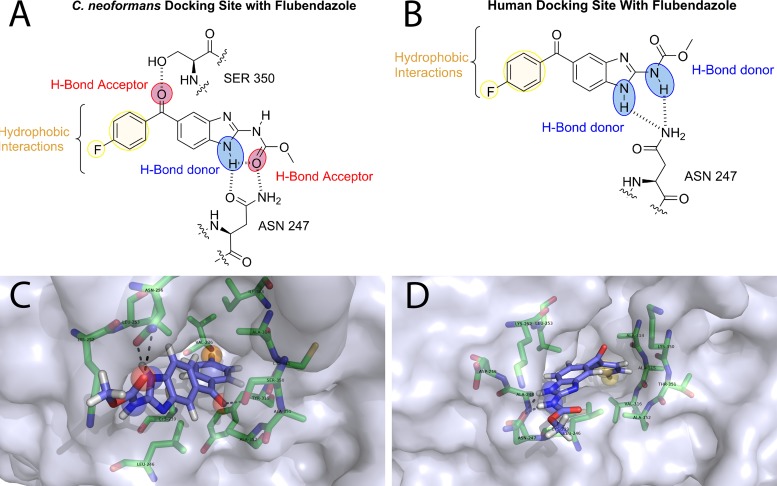
(A and B) Homology model of flubendazole docked with both C. neoformans (A) and human (B) β-tubulin. Red sphere, hydrogen bond donors; blue sphere, hydrogen bond acceptors; yellow sphere, hydrophobic interactions. (C and D) The docking pose is visualized with PyMOL. Protein is shown as a surface representation colored 40% transparent light blue. Flubendazole is represented as sticks composed of carbon (light blue), hydrogen (white), nitrogen (dark blue), oxygen (red), and fluorine (cyan). Binding site residues selected around 4 Å are represented as sticks with carbon (green), nitrogen (blue), oxygen (red), and sulfur (yellow).

There were two principal noncovalent binding interactions between flubendazole and the homology model of C. neoformans β-tubulin. First, the hydroxyl group of serine 350 (Ser350) acts as a hydrogen bond donor and binds the ketone oxygen of flubendazole (Fig. 1A). Second, asparagine 247 (Asn247) acted as a hydrogen bond donor via the primary amide with the ketone of the carbamate on flubendazole, but it also acted as a hydrogen bond acceptor through the primary carbonyl group of Asn247 and the N-H on the benzimidazole core. There were also several hydrophobic interactions deeper in the binding pocket that involved the benzene ring and the fluorine of flubendazole.

Docking studies of flubendazole and human β-tubulin (Fig. 1B) showed that both the N-H of the benzimidazole core and the N-H of the carbamate are hydrogen bond donors (Fig. 1B) to the primary amide of the side chain of Asn247. As for the C. neoformans interaction, there were hydrophobic interactions present from the *para*-substituted benzene and the hydrophobic binding pocket. There was a lack of a hydrogen bond acceptor role from the ketone oxygen. This is due to the replacement of Ser350 from the C. neoformans active site with lysine 350 (Lys350) in humans.

### Hollow-fiber infection model of cryptococcal meningoencephalitis.

**FIG 2:**
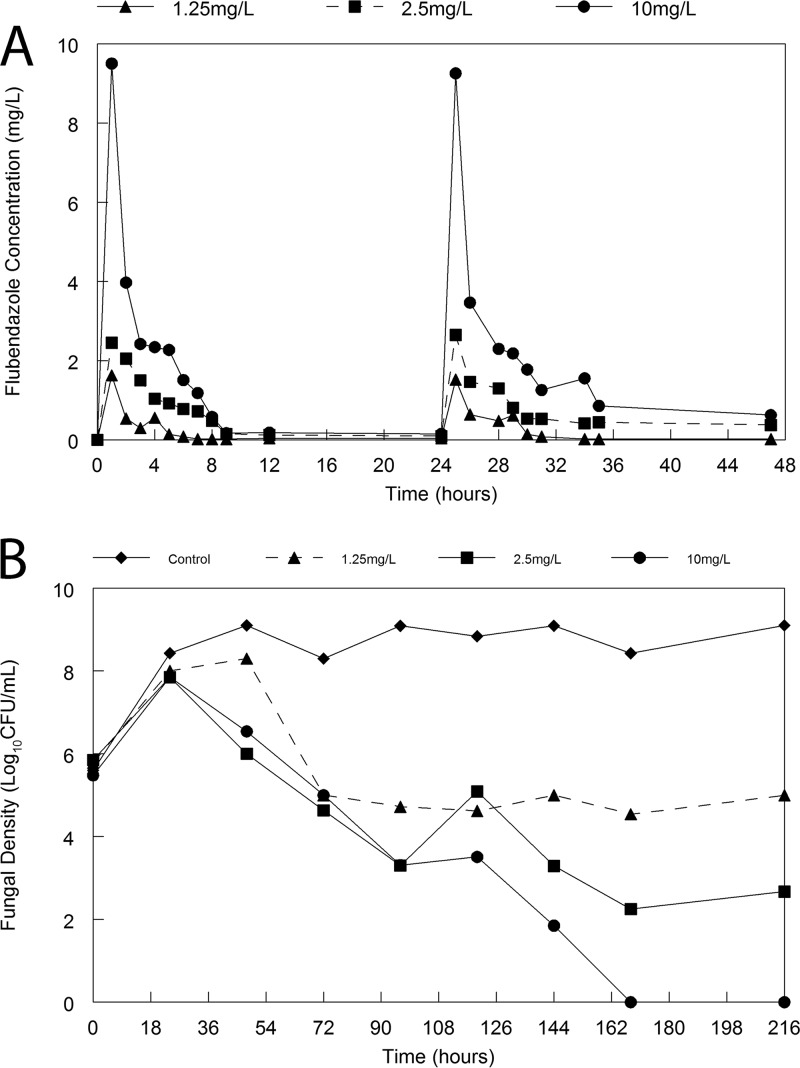
Hollow-fiber infection model of cryptococcal meningitis. (A) Pharmacokinetics of flubendazole with the three arms with intended peak concentrations of 1.25, 2.5, and 10 mg/liter; (B) pharmacodynamics in response to flubendazole administered at various doses q24h. Therapy was initiated 24 h postinoculation, after which time Cryptococcus had grown from ∼6 log_10_ CFU/ml to 8 log_10_ CFU/ml.

Rapid fungicidal activity was observed in the hollow-fiber infection model. Controls grew from an initial density of approximately 6 log_10_ CFU/ml to 8 to 9 log_10_ CFU/ml (Fig. 2). Following the administration of flubendazole there was a progressive decline in the fungal density in the hollow fiber in all arms. There was an exposure-dependent decline in fungal burden.

### Preliminary studies to demonstrate *in vivo* efficacy of flubendazole.

There was no demonstrable antifungal effect of orally administered flubendazole as a pure compound when formulated with sterile distilled water, 0.05% polysorbate 80 in phosphate-buffered saline (PBS), 5% dimethyl sulfoxide DMSO, 10% polyethylene glycol 400 (PEG400), or 85% hydroxyl-propyl-β-cyclodextrin (data not shown). Antifungal activity could be established only when pure flubendazole was formulated with polysorbate 80 (Tween 80) and injected subcutaneously (s.c.) to form a depot. Presumably, formulation with polysorbate 80 solubilized flubendazole to an extent that enabled it to become systemically bioavailable. However, this was observed only when flubendazole was administered s.c. This parenteral regimen resulted in a modest reduction in fungal burden, 1 to 2 log_10_ CFU/g, compared with that in vehicle-treated controls (data not shown). A limited PK study with concentrations measured at a single time point the end of the experiment also confirmed that flubendazole concentrations were quantifiable in plasma and the cerebrum of mice (data not shown).

These preliminary pharmacokinetic and pharmacodynamic data provided the impetus for further detailed experiments examining the pharmacodynamics of a new orally bioavailable solid drug nanodispersion against Cryptococcus neoformans developed by Janssen.

### Pharmacokinetic and pharmacodynamic studies of the flubendazole nanoformulation in mice.

**FIG 3:**
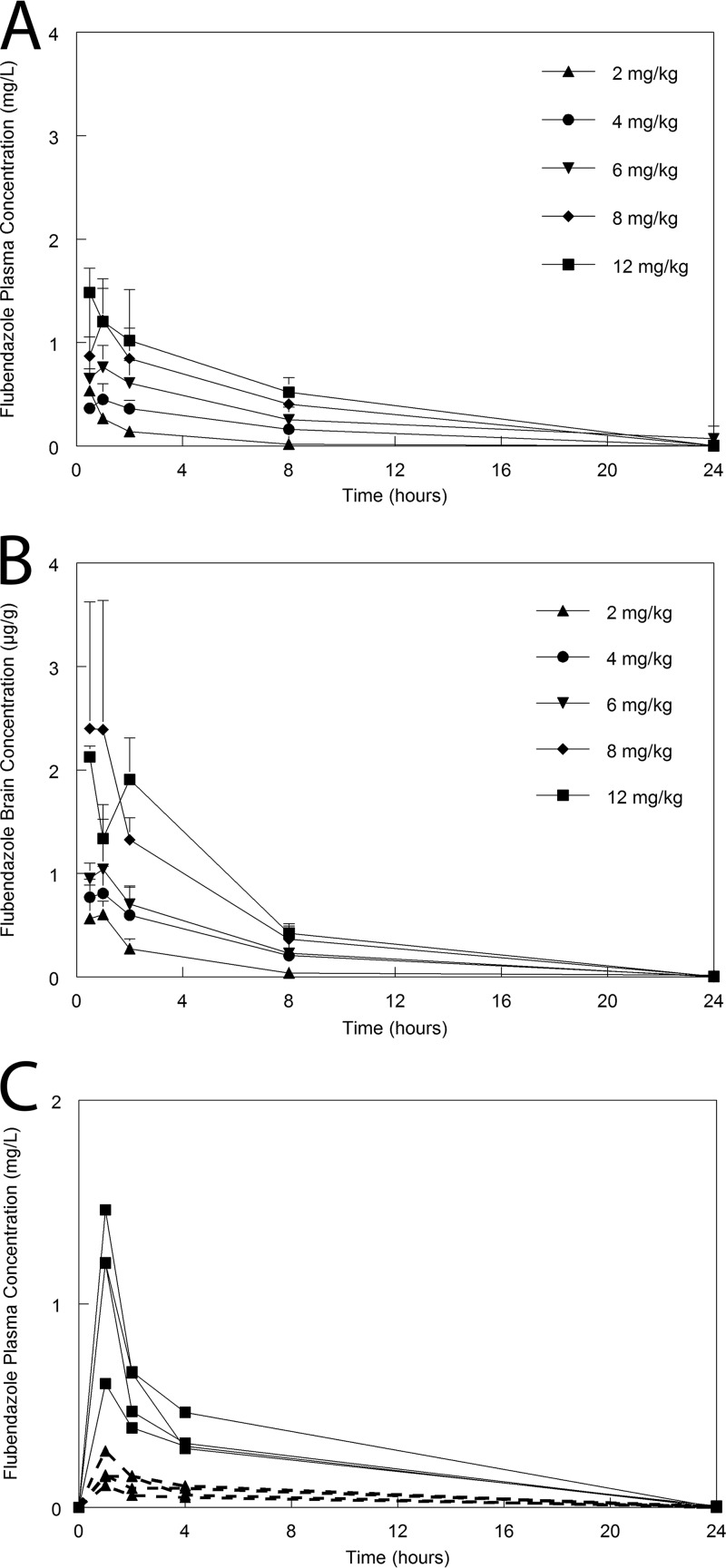
Flubendazole pharmacokinetics in mice and rabbits. (A) Mouse plasma concentration-time profiles following the administration of flubendazole at 2, 4, 6, 8, and 12 mg/kg. (B) Mouse concentration-time profiles in the brain following the administration of flubendazole at 2, 4, 6, 8, and 12 mg/kg. Data are means ± SDs for 3 mice. (C) Plasma pharmacokinetics in the serum for individual rabbits receiving 6 mg/kg/day (broken lines and solid triangles) and 22.5 mg/kg (solid lines and solid squares).

When flubendazole was formulated as a solid drug nanodispersion, it was rapidly absorbed after oral dosing and plasma concentrations were readily quantifiable at the first sampling point (i.e., 0.5 h postdose [Fig. 3]). The pharmacokinetics was linear, with biexponential clearance from the bloodstream with mean and median values of 0.039 and 0.026 liter/h, respectively (Fig. 3). The pharmacokinetic parameters are summarized in Table 2. There was rapid and extensive distribution of drug to the cerebrum in mice, and concentrations of flubendazole were consistently higher than those observed in plasma. The ratio of the area under the curve (AUC) for serum and the AUC for the cerebrum was 1:4.44.

**TABLE 2:** Parameter values from the PK-PD model fitted to mice

Parameter^*a*^	Mean	Median	SD
*K_a_* (h^−1^)	11.312	14.895	6.594
SCL/F (liters/h)	0.039	0.026	0.031
*V_c_*/F (liters)	0.051	0.069	0.033
*K*_cp_ (h^−1^)	15.741	15.404	6.806
*K*_pc_ (h^−1^)	16.997	16.915	5.962
*K*_cb_ (h^−1^)	3.446	0.594	4.709
*K*_bc_ (h^−1^)	0.056	0.056	0.030
*K*_gmax_ (log_10_ CFU/g/h)	0.107	0.098	0.025
Hg	10.338	5.096	9.782
*C*_50g_ (liters/h)	2.036	1.681	1.517
POPMAX (CFU/g)	982,934,669.178	427,055,621.187	2,281,967,059.602
IC (CFU/g)	102.255	116.462	60.966
*V_b_*/F (liters)	0.277	0.146	0.335

a *K_a_* is the first-order rate constant collecting the gut and the central compartment; SCL/F is the apparent clearance of flubendazole from the central compartment; *V_c_*/F and *V_b_*/F are the apparent volumes of the central compartment and brain, respectively; *K*_cp_, *K*_pc_, *K*_cb_, and *K*_bc_ are the first-order intercompartmental rate constants; and *K*_gmax_ is the maximal rate of cryptococcal growth. POPMAX is the maximum theoretical fungal density. *C*_50g_ is the concentrations of flubendazole that induce half-maximal effects on growth. Hg is the slope function for the effect of flubendazole on growth. IC is the density of *Cryptococcus* immediately postinoculation.

**FIG 4:**
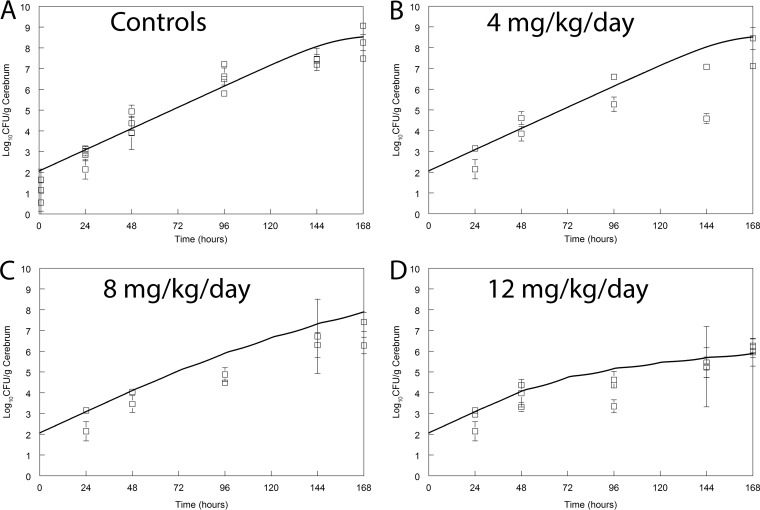
Pharmacodynamics of flubendazole in a murine model of cryptococcal meningitis. Flubendazole was administered orally once daily. Data (open squares) are means ± SDs for 3 mice. The solid line is the fit of the population predicted pharmacokinetic-pharmacodynamic model. The maximally administered dose in this study (12 mg/kg/day) slowed but did not prevent fungal growth in the brain.

Flubendazole had a significant and discernible antifungal effect in mice. Use of the highest dose in this study (12 mg/kg) resulted in approximately a 2- to 3-log reduction in fungal burden relative to that of controls (Fig. 4). This regimen was limited by maximum permissible volumes for oral administration for mice (i.e., 20 ml/kg). In a single experiment in which the effect of 6 mg/kg every 12 h (q12h; i.e., 12 mg/kg/day) was compared to that of 12 mg/kg/day, there was no difference in antifungal effect (data not shown). This is preliminary evidence that the AUC is likely to be the dynamically linked index for flubendazole against Cryptococcus neoformans.

### Pharmacokinetic and pharmacodynamic studies with rabbits.

The PK in rabbits was linear, with a concentration-time profile similar to that observed in mice. The plasma concentration-time profiles in rabbits had a similar shape to those of mice but were lower for the dosages used in this study. Despite readily quantifiable plasma concentrations, there were no quantifiable drug concentrations in either the cerebrospinal fluid (CSF) or the cerebrum in rabbits at the time of sacrifice.

**FIG 5:**
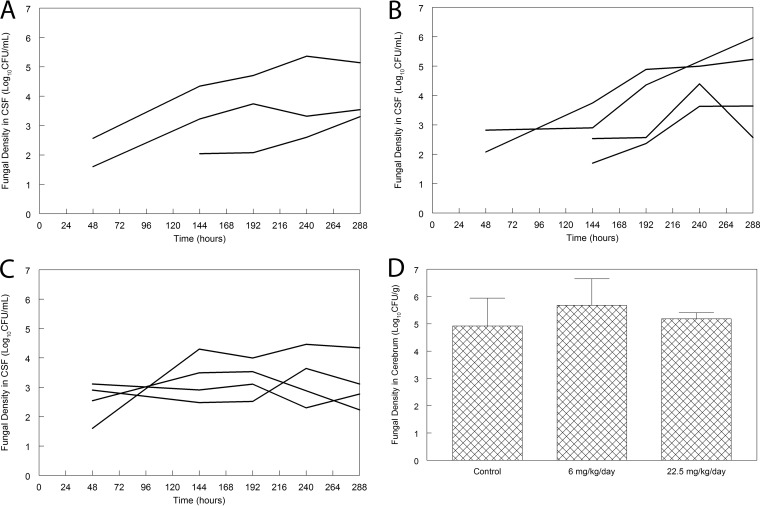
Pharmacodynamics of flubendazole in a rabbit model of cryptococcal meningitis. (A) Time course of fungal burden in the CSF of untreated controls; (B) time course of fungal burden in the CSF rabbits treated with flubendazole at 6 mg/kg q24h orally; (C) time course of fungal burden in the CSF rabbits treated with flubendazole at 22.5 mg/kg q24h orally; (D) fungal burden in the cerebrum of rabbits at the end of the experiment (288 h postinoculation and after 10 days of treatment with flubendazole). There are no differences in the three groups (*P* = 0.464, analysis of variance).

There was no demonstrable antifungal effect in rabbits receiving 6 mg/kg/day. There may have been some effect in rabbits receiving 22.5 mg/kg q24h, but if present, the effect was small, and these assessments were limited by the use of few animals. There were no statistically significant differences in the area under the concentration (log_10_ CFU/g)-time curve for each regimen, even though this may be a relatively insensitive test of antifungal effect. Furthermore, there was no difference in the fungal burden in the cerebrum at the end of the experiment for any of the groups of rabbits used in this study (Fig. 5).

## DISCUSSION

When given subcutaneously, flubendazole has striking activity in laboratory animal models of filarial diseases such as onchocerciasis and lymphatic filariasis (16). Janssen developed a novel amorphous solid drug nanodispersion to provide a potential new therapeutic option for patients with these neglected tropical diseases. The systemic drug exposure that was enabled by the new formulation mandated Good Laboratory Practice (GLP) toxicology studies before progression to early-phase clinical studies. It was already known that flubendazole is clastogenic (i.e., induces chromosomal breakages) and aneugenic (i.e., induces aneuploidy), as well as embryotoxic (17). GLP toxicology studies were performed by Janssen with rats (5, 15, and 30 mg/kg/day in male rats and 2.5, 5, and 10 mg/kg/day in female rats) and dogs (20, 40, and 100 mg/kg/day) for 2 weeks. These experiments showed evidence of toxicity related to the pharmacological activity of flubendazole in the gastrointestinal tract, lymphoid system, and bone marrow, as well as testicular toxicity in both rats and dogs. In dogs, liver toxicity was also observed. As a result, the development program was stopped based on an unacceptable risk/benefit profile in humans. This also halted our own efforts to develop flubendazole for cryptococcal meningitis.

Flubendazole has striking *in vitro* activity against Cryptococcus neoformans that was evident in the MIC testing and the pharmacodynamic studies in the hollow-fiber infection model. There was modest antifungal activity in the murine model, which is not as prominent as that previously described by us for fluconazole, amphotericin B deoxycholate, or liposomal amphotericin B (8, 18, 19). There was no unequivocal antifungal activity in the rabbit model of cryptococcal meningoencephalitis, which is largely explained by the absence of detectable flubendazole concentrations in the cerebrum or CSF (despite readily quantifiable plasma concentrations). The *in vitro* susceptibility testing and data from the hollow-fiber model suggest that flubendazole is highly potent and fungicidal if able to reach its fungal target in sufficient concentrations. The diminished activity in mice (relative to historical controls) and absence of effect in rabbits (with nonquantifiable concentrations in the cerebrum and CSF) further support this conclusion. Hence, successful exploitation of the benzimidazole backbone requires careful attention to physiochemical properties that promote absorption across the gut and the ability to partition into subcompartments of the central nervous system (CNS).

Flubendazole did not display a degree of *in vivo* activity comparable to those of other first-line agents for cryptococcal meningitis (i.e., fluconazole and amphotericin B formulations). Even if the safety profile was not problematic, there is insufficient prima facie evidence from either the murine or rabbit model to further study flubendazole as monotherapy for induction therapy in phase II clinical studies. Nevertheless, additional approaches, such as combination with other antifungal agents for induction therapy and/or use as longer-term consolidation and maintenance therapy, may have been possible.

The potential of derivatives of flubendazole to be useful human medicines depends on the differential activity between cryptococcal and human proteins. Characterization of the β-tubulin genes of C. neoformans has been undertaken, and two C. neoformans β-tubulin genes (*TUB1* and *TUB2*) have been identified. *TUB1* was identified as the primary target of the benzimidazole class of compounds through gene characterization and expression (14). There is 90% homology between fungal *TUB1* and human β-tubulin, although the former has not been crystallized and this has prevented definitive structure-activity-relationship docking studies. The ability to develop new agents based on a benzimidazole scaffold or to further exploit β-tubulin as a pharmacological target will depend on the degree of differential activity of a benzimidazole with these proteins. The differential binding identified through the docking and homology modeling of both human β-tubulin (20) and C. neoformans var. *grubii* serotype A (strain H99) β-tubulin (14) to the Bos Taurus 1SA0 β-tubulin crystal structure implies an increased number of binding interactions with C. neoformans β-tubulin. This may provide the potential to exploit this differential binding to establish a favorable therapeutic index. It is also worth emphasizing that the benzimidazoles may have additional targets beyond β-tubulin that have the further potential to provide differential activity between human and fungal proteins, but this requires further investigation (21–24).

The potential utility of congeners of flubendazole now rests with medicinal chemistry programs. Compounds must be synthesized that exhibit differential activity against cryptococcal and human tubulin (if that is possible) so that there is an acceptable safety margin and toxicity profile. Furthermore, the compound must be able to traverse the gut (compounds that are not orally bioavailable will be less clinically valuable) and then the blood-brain barrier to achieve concentrations that are ideally fungicidal. The latter will be promoted by new low-molecular-weight, lipophilic compounds that are not substrates for efflux pumps such as P-glycoprotein. This will undoubtedly also require the use of novel formulation technologies to ensure that compounds that are poorly soluble can become useful agents for disseminated infections.

## MATERIALS AND METHODS

### Drug.

Flubendazole that was used for determination of MICs, hollow-fiber experiments, and preliminary murine experiments was purchased from Sigma. Subsequently, definitive pharmacokinetic-pharmacodynamic experiments that were performed with orally administered flubendazole used a solid drug nanodispersion formulation developed by Janssen Pharmaceuticals (batch BREC-1113-070; Janssen Pharmaceuticals). The stability of this formulation in liquid and solid phases was confirmed for 1 and 6 months, respectively.

A 10-mg/ml methylcellulose (4,000-cps) stock solution (100-ml batch) was prepared from a dispersion of 1 g of methylcellulose (4,000 cps) with stirring into 70 ml of demineralized water heated to 70°C to 80°C. The solution was stirred for at least 15 min, followed by the addition of 20 ml of demineralized water. The mixture was stirred until it reached room temperature and was then made up to 100 ml with demineralized water. A total of 50 ml of 6-mg/ml spray-dried powder suspension was then prepared (this corresponds to 0.6 mg/ml of flubendazole as the active dose in 0.5% methylcellulose). A total of 24.70 g of demineralized water was added to a 50-ml clear glass vial. A total of 0.30 g of spray-dried drug was added to the vial, which was then closed with a stopper. The vial was vortexed and then homogenized using a Polytron disperser. A 25-ml stock solution of methylcellulose (4,000 cps) was added and the vial was vortexed. The suspension was refrigerated at 5°C until dosing for a maximum of 14 days. Prior to dosing, the suspension was vortexed.

### Strains.

The initial *in vitro* susceptibility testing was performed with H99 (ATCC 208821). An additional 49 clinical isolates were obtained from the National Centre for Microbiology Instituto de Salud Carlos III, Madrid, Spain (courtesy of Ana Alastruey-Izquierdo and Manual Cuenca-Estrella). These isolates were identified to the species level using standard microbiological techniques.

### MICs.

The MIC of flubendazole against H99 (ATCC 208821) and the 49 isolates was estimated using methodology of the European Committee on Antimicrobial Susceptibility Testing (EUCAST [25]) and the Clinical and Laboratory Standards Institute (CLSI [26]). The endpoint for MIC determination using was 50% for both the EUCAST and CLSI methods. MICs were performed in triplicate.

### Porcine tubulin polymerization assay.

Porcine tubulin is generally used as a surrogate for human tubulin because of its high degree of homology (95%) (27). In the studies described here, this assay was used to determine the extent of interaction between flubendazole and its putative target; it has also been used for assessment of the binding of antineoplastic agents (28, 29). The commercially available porcine tubulin assay (BK011P; Cytoskeleton, Inc., Denver, CO) quantifies the time-dependent polymerization of tubulin to microtubules and thus the ability of tubulin inhibitors to disrupt this process.

The porcine tubulin assay was performed according to the manufacturer's instructions. Briefly, the 96-well assay plate was prewarmed to 37°C prior to use. Five microliters of test compound(s) and controls at 0, 1.25, 2.5, 5, and 10 μM were aliquoted into each well and prewarmed for 1 min. Colchicine and DMSO were used as positive and negative controls, respectively. Polymerization was initiated by mixing 45 μl of reaction buffer that contained 2 mg/ml of purified porcine brain tubulin, 10 μM fluorescent reporter, PEM buffer (80 mM PIPES, 0.5 mM EGTA, 2 mM MgCl_2_ [pH 6.9]), 1 mM GTP, and 20.3% glycerol. Tubulin polymerization was followed by an increase in fluorescence intensity due to the incorporation of a fluorescence reporter into microtubules as polymerization occurred. The change in fluorescence was measured using an excitation and emission wavelength of 360 nm and 450 nm, respectively, every 1 min for 1 h at 37°C using a Varioskan multimode plate reader (Thermo scientific Inc.). All data points were acquired in triplicate, and IC_50_s were calculated with GraphPad Prism. The IC_50_ was defined as the drug concentration required to inhibit tubulin polymerization by 50% compared with the negative control.

### Homology modeling and docking studies.

While the amino acid sequence of cryptococcal β-tubulin is known (74% homology with human β-tubulin), the protein has not been crystallized. A homology model was therefore developed to investigate differential binding modes of flubendazole within C. neoformans and human β-tubulin. Molecular modeling (Modeler version 9.14; https://salilab.org/modeller/) of both human β-tubulin (20) and C. neoformans var. *grubii* serotype A (strain H99) β-tubulin (14) was undertaken using the Bos Taurus 1SA0 β-tubulin crystal structure (identity, 364/447 [81.4%]; similarity, 405/447 [90.6%]).

Virtual flubendazole was built in the molecular modeling software Spartan (Wavefunction Inc., Irvine, CA) and energy minimized. Flubendazole was then subjected to a piecewise linear potential (ChemPLP) docking protocol (a scoring function to provide confidence in the docking pose adopted by the molecule), consisting of 10 genetic algorithm (GA) runs before visualization using the molecular visualization system PyMOL with the top scoring compound depicted in Fig. 1. The active-site binding interactions were selected by identifying those amino acid residues within 4 Å of flubendazole when docked into the β-tubulin binding site. Polar contacts between flubendazole and the surrounding amino acids were identified, which aided in the identification of hydrogen bonding interactions that are key in determining the efficacy of a drug against its pharmacological target.

Finally, hydrogen bond donor interactions as well as hydrophobic interactions were identified using the pharmacophore (i.e., an abstract description of molecular features that in this case are necessary for molecular recognition of flubendazole by β-tubulin) search software ZINCPharmer (30) at 4 Å for hydrogen bonding interactions and 6 Å for hydrophobic interactions.

### Hollow-fiber model of cryptococcal meningoencephalitis.

A new hollow-fiber infection model was developed to investigate the *in vitro* pharmacodynamics of flubendazole against C. neoformans. The same cartridges (FiberCell Systems, Frederick, MD) and configuration as previously described for bacterial pathogens was used (see, for example, reference 31). The extracapillary space of each cartridge was inoculated with 40 ml of a suspension containing 6 log_10_ CFU/ml of C. neoformans var. *grubii* (ATCC 208821; H99). Yeast extract-peptone-dextrose (YPD) medium was pumped from the central compartment through the cartridge and back again using a peristaltic pump (205 U; Watson-Marlow, United Kingdom). The hollow-fiber infection model was incubated at 37°C in ambient air. The time course of fungal growth was determined by removing 1 ml from the extracapillary space of the cartridge and plating serial 10-fold dilutions to YPD agar.

The relationship between flubendazole exposure and its effect was explored using a range of drug exposures. Since there is no information on the pharmacokinetics of flubendazole in humans, we attempted to produce AUCs that were comparable to those observed in mice. Various doses of flubendazole were administered q24h by infusion over 1 h for 8 days to the central compartment using a programmable syringe driver (Aladdin pump; World Precision Instruments, United Kingdom). There was a 24-h delay in the initiation of flubendazole therapy postinoculation. To generate first-order pharmacokinetics, fresh YPD medium was pumped into the central compartment and the same volume of drug-containing medium was simultaneously removed and discarded. Positive controls of currently licensed agents were not studied in these experiments.

### Murine model of cryptococcal meningoencephalitis.

A previously described (8) and well-characterized murine model of cryptococcal meningitis was used to investigate the pharmacodynamics of flubendazole. All laboratory animal experiments were performed under UK Home Office project license PPL40/3630 and were approved by the University of Liverpool's Animal Welfare Ethics Review Board. Male CD1 mice were purchased from Charles River and were 20 to 30 g at the time of experimentation. An inoculum of 3 × 10^8^ CFU in 0.25 ml was used for each mouse. Groups of mice (*n* = 3) were serially sacrificed throughout the experimental period. The brains were removed and homogenized. Serial 10-fold dilutions were plated to YPD agar supplemented with chloramphenicol to enumerate the total fungal burden. Plates were incubated in air at 30°C for at least 48 h.

### Studies of pharmacokinetics and pharmacodynamics of flubendazole in mice.

Preliminary evidence for the efficacy of flubendazole was obtained by dissolving pure compound in a variety of excipients that included cyclodextrin (F2G; Eccles, UK), DMSO (5%), and polysorbate 80 (10%) and injecting it subcutaneously q24h. Ultimately, only s.c. injection with Tween 80 showed any effect. This experiment provided the impetus to further examine the orally bioavailable formulation developed by Janssen (see above).

The pharmacokinetics of oral flubendazole was determined with two independently conducted experiments. Treatment was initiated 24 h postinoculation. Doses of 2 to 12 mg/kg were used. Only the first dosing interval was studied. A serial-sacrifice design was used with groups of 3 mice that were sacrificed at 0.5, 1, 2, 8, and 24 h postinoculation.

The pharmacodynamics of oral flubendazole was estimated over the course of three separate independently conducted experiments. Groups of 3 mice were sacrificed at 2, 24, 48, 96, 144, and 168 h postinoculation. Dose finding studies were performed using flubendazole at 2, 4, 6, 8, and 12 mg/kg q24h orally. The upper dosage was limited by the volume restrictions for mice imposed by Home Office project license PPL40/3630. A fourth experiment compared 12 mg/kg q24 with 6 mg/kg q12h to examine whether more fractionated regimens provided any additional antifungal effect.

### Rabbit model of cryptococcal meningitis.

A previously described and well-characterized rabbit model of cryptococcal meningoencephalitis (32) was used to further investigate the pharmacodynamics of flubendazole. Male New Zealand White rabbits were purchased from Harlan. Rabbits weighed 2.5 to 3 kg at the time of experimentation. Rabbits were immunosuppressed intramuscularly with hydrocortisone at 10 mg/kg on day −1 relative to infection and then daily throughout the experiment.

Cryptococcal meningoencephalitis was induced with the intracisternal inoculation of 0.25 ml of a suspension containing 3.8 × 10^8^ CFU/ml under general anesthesia (induced with medetomidine and ketamine). This inoculum results in progressive infection that manifests as an increase in fungal burden in the CSF and reproducible encephalitis. There was minimal clinical disease with no demonstrable neurological signs in the experimental period. Mortality always occurred in the context of cisternal tapping and repeated anesthesia rather than from progressive infection.

### Studies of pharmacodynamics and pharmacokinetics in rabbits.

PK-PD relationships in the rabbit were estimated in two independently conducted experiments including 6 rabbits in each. Rabbits were placed under general anesthesia for removal of CSF via intracisternal tapping at 48 h intervals. Over the course of the two experiments there were 3 controls (1 rabbit died after being tapped); flubendazole was given at 6 mg/kg q24h (*n* = 6) and 22.5 mg/kg q24h (*n* = 6). The maximum dosage that was used was limited by the formulation provided by Janssen and the limits of oral gavage in rabbist (15 ml/kg/day). Treatment was initiated 48 h postinoculation and continued for 10 days, after which time all rabbits were sacrificed. Thus, the total duration of the experiment was 288 h.

### Measurement of flubendazole concentrations using LC-MS/MS.

Flubendazole concentrations in all matrices were measured using validated ultrahigh-performance liquid chromatography-tandem mass spectrometry (LC-MS/MS) implemented on an Agilent 6420 triple quad mass spectrometer and an Agilent 1290 infinity LC system (Agilent Technologies UK Ltd., Cheshire, UK). Flubendazole was extracted by protein precipitation by adding 300 μl of a 50:50 mix of acetonitrile and methanol that contained the internal standard [6,7-dimethyl-2,3-di(2-pyridyl) quinoxaline; Sigma-Aldrich, Dorset, UK] at a final concentration of 1 mg/liter to 30 μl of each matrix.

The extraction was performed in 96-well Sirocco protein precipitation plates (Waters, UK). Samples were then shaken for 2 min and extracted using a positive-pressure 96 manifold (Waters). A total of 200 μl of the supernatant was removed and placed in a 96-well plate. One microliter was injected on an Agilent a Zorbax Eclipse Plus C_18_ column (2.1 by 50 mm; 1.8-μm particle size; Agilent Technologies UK Ltd.). Chromatographic separation was achieved using a gradient with the starting conditions of a 60:40 mix of A (0.1% formic acid in water) and B (0.1% formic acid in acetonitrile). The ratio of A to B changed to 20:80 over 2 min and then returned to the starting conditions (60:40) for 1 min of equilibration.

The mass spectrometer was operated in multiple reaction monitoring (MRM) scan mode in positive polarity. The precursor ions for flubendazole and the internal standard were 314.1 *m/z*, and 313.15 *m/z*, respectively. The product ions for flubendazole and the internal standard were 282.1 *m/z* and 284.1 *m/z*, respectively. The source parameters were set as 4,000 V for capillary voltage, 350°C for gas temperature, and 60 lb/in^2^ for the nebulizer gas.

The standard curve for flubendazole encompassed the concentration range of 0.0005 to 8.0 mg/liter and was constructed using the respective blank matrix. The limit of quantitation was 0.0005 mg/liter, the coefficient of variation (CV) was 12.7% over the concentration range of 0.0005 to 8 mg/liter, and the intra- and interday variation was <12% for all matrices.

### Mathematical modeling.

The pharmacokinetic and pharmacodynamic data sets from mice were modeled using the program Pmetrics (33) and the following five inhomogeneous differential equations:

(1) $$XP(1)=B(1)-K_{a}\times X\left(1\right)$$

(2) $$XP(2)=K_{a}\times X(1)-(\frac{\text{SCL}}{V})\times X(2)-K_{\text{cp}}\times X(2)+K_{\text{pc}}\times X(3)-K_{\text{cb}}\times X(2)+K_{\text{bc}}\times X\left(4\right)$$

(3) $$XP(3)=K_{\text{cp}}\times X(2)-K_{\text{pc}}\times X\left(3\right)$$

(4) $$XP(4)=K_{\text{cp}}\times X(2)-K_{\text{pc}}\times X\left(4\right)$$

(5) $$XP\left(5\right)\;=\;K_{\text{gmax}}\;[1-\left(\frac{{\left(\frac{X\left(4\right)}{V}\right)}^{\text{Hg}}}{{C_{50\text{g}}}^{\text{Hg}}+{\left(\frac{X\left(4\right)}{V}\right)}^{\text{Hg}}}\right)]\;\times \;[1-\left(\frac{X\left(5\right)}{\text{POPMAX}}\right)]\times X\left(5\right)$$

The system parameters and their units are as follows: *B*(*1*) (mg) represents the bolus input of flubendazole into the gut. *K_a_* (h^−1^) is the first-order rate constant collecting the gut and the central compartment, SCL (liters/h) is the clearance of flubendazole from the central compartment, *V* (liters) is the volume of the central compartment, and *K*_cp_ (h^−1^) and *K*_pc_ (h^−1^) are the first-order intercompartmental rate constants. *K*_gmax_ (log_10_ CFU/g/h) is the maximal rate of cryptococcal growth. POPMAX (CFU/g) is the maximum theoretical fungal density. *C*_50g_ (mg/liter) is the concentration of flubendazole that induces half-maximal effects on growth. Hg is the slope function for growth. The initial condition (CFU/g; not shown in the equations) is the fungal density immediately following inoculation and is estimated along with other parameters.

Equations 1, 2, 3, and 4 are pharmacokinetic equations that describe the movement of drug from the gut, throughout the body, and into the brain. Equation 1 describes the movement of drug from the gut. Equation 2 describes the rate of change of flubendazole in the central compartment (plasma) with first-order clearance and movement of drug to and from both a peripheral (unmeasured) compartment and the cerebrum. Equations 3 and 4 describe the rate of change of drug in the peripheral and cerebral compartments, respectively. The pharmacodynamics of flubendazole against Cryptococcus neoformans is described by equation 5, which has terms that describe the capacity-limited growth of Cryptococcus and flubendazole-induced suppression of growth. The antifungal activity in the cerebrum is primarily related to concentrations in the cerebrum.

A similar model was used to model the PK-PD data from rabbits, but there were some differences. First, no drug was detectable in the brain or the CSF. Therefore, we let plasma concentrations of drug drive the antifungal effect and did not attempt to model the concentration of drug in the central nervous system (as was the case for mice). We directly linked plasma concentrations with the antifungal effect.
